# Characterization of salt tolerant wheat genotypes by using morpho-physiological, biochemical, and molecular analysis

**DOI:** 10.3389/fpls.2022.956298

**Published:** 2022-08-22

**Authors:** Ahsan Irshad, Rana Imtiaz Ahmed, Shoaib Ur Rehman, Guozhong Sun, Furqan Ahmad, Muhammad Ali Sher, Muhammad Zahid Aslam, Mohamed M. Hassan, Sameer H. Qari, Muhammad Kashif Aziz, Zulqurnain Khan

**Affiliations:** ^1^National Engineering Laboratory of Crop Molecular Breeding, National Center of Space Mutagenesis for Crop Improvement, Institute of Crop Sciences, Chinese Academy of Agricultural Sciences, Beijing, China; ^2^Regional Agricultural Research Institute, Bahawalpur, Pakistan; ^3^Ayub Agricultural Research Institute, Faisalabad, Pakistan; ^4^SINO-PAK Joint Research Laboratory, Institute of Plant Breeding and Biotechnology, Muhammad Nawaz Sharif University of Agriculture, Multan, Pakistan; ^5^National Engineering Research Center of Crop Molecular Breeding, Institute of Crop Sciences, Chinese Academy of Agricultural Sciences, Beijing, China; ^6^Cotton Research Station, Bahawalpur, Pakistan; ^7^Department of Biology, College of Science, Taif University, Taif, Saudi Arabia; ^8^Department of Biology, Al-Jumum University College, Umm Al-Qura University, Makkah, Saudi Arabia

**Keywords:** salt tolerance, sodium chloride, wheat yield, expression analysis, high affinity potassium transporters

## Abstract

Food security is facing a major threat from salinity and there is a need to develop salt tolerant crop varieties to ensure that the demand for food from the world’s increasing population is met. Salinity mostly occurs in arid and semi-arid regions. It may cause many adverse physiological effects on plants, i.e., toxic ion accumulation, disturbed osmotic potential, and decreased crop yield. The present study aimed to investigate the morphological, physiological, biochemical, and genetic parameters of wheat genotypes under salt stress. Six wheat genotypes were screened for salt tolerance at the seedling and maturity stage. Seeds were sown at 0 and 150 mM of salinity level. Biochemical traits, i.e., shoot/root fresh and dry weight, chlorophyll a/b and total chlorophyll contents, shoot nitrogen, shoot phosphorus, proline, and carbohydrates were measured. Wheat genotypes showed a significant increase in free amino acids, shoot nitrogen, and total soluble proteins under saline conditions. Higher Na^+^/K^+^ ratio and free amino acids were estimated under 150 mM NaCl treatment in Pasban-90 and found to be the most salt-tolerant genotype. By contrast, reduced proline, total chlorophyll, and Na^+^/K^+^ ratio were found in Kohistan-97 marking it to be sensitive to stress. Expression analysis of *HKTs* genes was performed to validate the results of two contrasting genotypes. The differential expression of *HKT2; 1* and *HKT2; 3* explained the tissue and genotype specific epigenetic variations. Our findings indicated that these selected genotypes can be further used for molecular studies to find out QTLs/genes related to salinity. This suggests that, in contrasting wheat genotypes, there is a differentially induced defense response to salt stress, indicating a functional correlation between salt stress tolerance and differential expression pattern in wheat.

## Introduction

Wheat is one of the most important cereal crops, providing 20% protein and 4.5 billion calories to people worldwide on a daily basis (Kiani et al., 2021). Wheat productivity is limited by environmental fluctuations including water, heat, cold, and salt stresses. Breeding for stress resistant crop germplasm is important for the sustainable production of food commodities. At present, salinity is becoming a worldwide problem because of its harmful effects on wheat growth and productivity. On a global scale, ~405 million hectares of land is saline, which is approximately one third of the world’s irrigated agricultural land (Munns, 2002; Sairam et al., 2002). Like many other parts of the world, salinity has become a great problem for Pakistan. In Pakistan, the increase in food production is not at par with the population, which poses a threat to food security (Azeem et al., 2019). Therefore, wheat breeding for salt stress resilience is a subject of interest that breeders have investigated in relation to the response of plants under stress conditions. For the identification of salt tolerant genotypes from wheat germplasm, there is a need to study the physiological, biochemical, and epigenetic changes in plants (Singh et al., 2015; Kumar et al., 2017). With better knowledge of these traits, researchers will be able to clone the genes involved in salt tolerance (Sairam et al., 2002).

Soil conditions are described as having salinity when excessive amounts of neutral soluble salts (including the sulphates and chlorides of Mg, K, Ca, and Na) are accumulated. Excessive amounts of these salts in soil or water cause adverse effects on the growth of plants by disrupting the uptake of essential macro-or micro-nutrients (Munns, 2002; Ashraf, 2004; Athar and Ashraf, 2009). Salinity results in a reduction of the yield, plant biomass, photosynthetic activities, transpiration rates, and osmotic potential, making wheat vulnerable to diseases. Furthermore, it may also result in the sequestration of toxic ions in the leaves and roots of crop plants (Ashraf and O’Leary, 1995). Accumulation of these ions in roots may lead to osmotic stress which eventually disturbs the cell ion balance by disrupting the normal uptake of essential nutrients, thus leading to the condition of nutrient deficiency. Accumulation of Na^+^ (Sodium ion) is one of the harmful effects of salinity. The accumulation of Na^+^ in the plant inhibits the intake of other essential macronutrients such as K^+^ (potassium ion) and Ca^+^ (calcium ion) from the soil (Brini et al., 2009). Whereas K^+^ is important for plant growth and development with the maintenance of the K^+^/Na^+^ ratio in the shoot, which is one of the main strategies to control stress in plants (Hamamoto et al., 2015). The chemical properties of Na^+^ and K^+^ are almost the same in the plant with the same content ratio in the non-saline soil but their physiological impact during the metabolism and growth of the plant is significantly different. Salinity-induced osmotic stress inhibits the photosynthesis of plants and causes changes in chlorophyll contents and other components. It also causes inhibition of photochemical activities and decreases the activities of enzymes in the Calvin cycle. The most remarkable event in leaf senescence is the disassembled functioning of the photosynthetic apparatus in chloroplasts, leading to a concomitant decrease in photosynthetic activity (Zhang et al., 2014). Thus, osmotic stress usually causes a decrease in crop production (Marcińska et al., 2013). Different physiological traits are considered positive indicators (Proline, shoot-root biomass soluble sugar etc.) in understanding the salt tolerance ability of plants. The only amino acid that acts as a good osmolyte is known as proline and this serves as a metal chelator, antioxidative defense molecule, and signaling molecule. It helps to prevent an oxidative burst in the plant by maintaining the osmotic balance and membrane integrity. The production of phenolic compounds plays an important role in antioxidant activities to neutralize free radicals and decompose peroxidase in plants. Biotic and abiotic stress initiated the accumulation of phenolic compounds.

Some transporters are active in the plasma membrane with Na^+^ uniporter as well and function as Na^+^/K^+^ symporters, known as high affinity potassium transporters (*HKTs*; Horie et al., 2009). Based on phylogenetic analyses, two families of HKTs (HKT1 and HKT2) have been reported. The transporters of the HKT1;x subfamily are permeable to Na^+^ only while another sub family (HKT2;y) is permeable to both Na^+^ and K^+^ (Platten et al., 2006). Under salt stress, the *TaHKT2* gene is down-regulated in salt-tolerant varieties (Singh et al., 2015). There is still a need to understand the molecular mechanism of wheat in gene regulation, which is equally important to increasing salt tolerance (Arzani and Ashraf, 2016).

Different findings have examined resistant varieties for salt tolerance in wheat using different levels of salt stress and evaluating the different traits related to salinity. Envirotype makes it much more complicated to screen for salt tolerant wheat germplasm in the salt affected field (Arzani, 2008; Singh et al., 2015). To assess the wheat germplasm’s ability to survive under salt stress conditions, the morphological, physiological, biochemical, and genetic parameters need to be examined. Several studies have revealed that wheat is moderately tolerant against salinity stress and improves this ability as the plant grows (Saqib et al., 2005). It has been reported that flowering and grain filling stages in wheat are more tolerant to salt as compared to seedling or vegetative stages (Impa et al., 2019).

**Figure 1:**
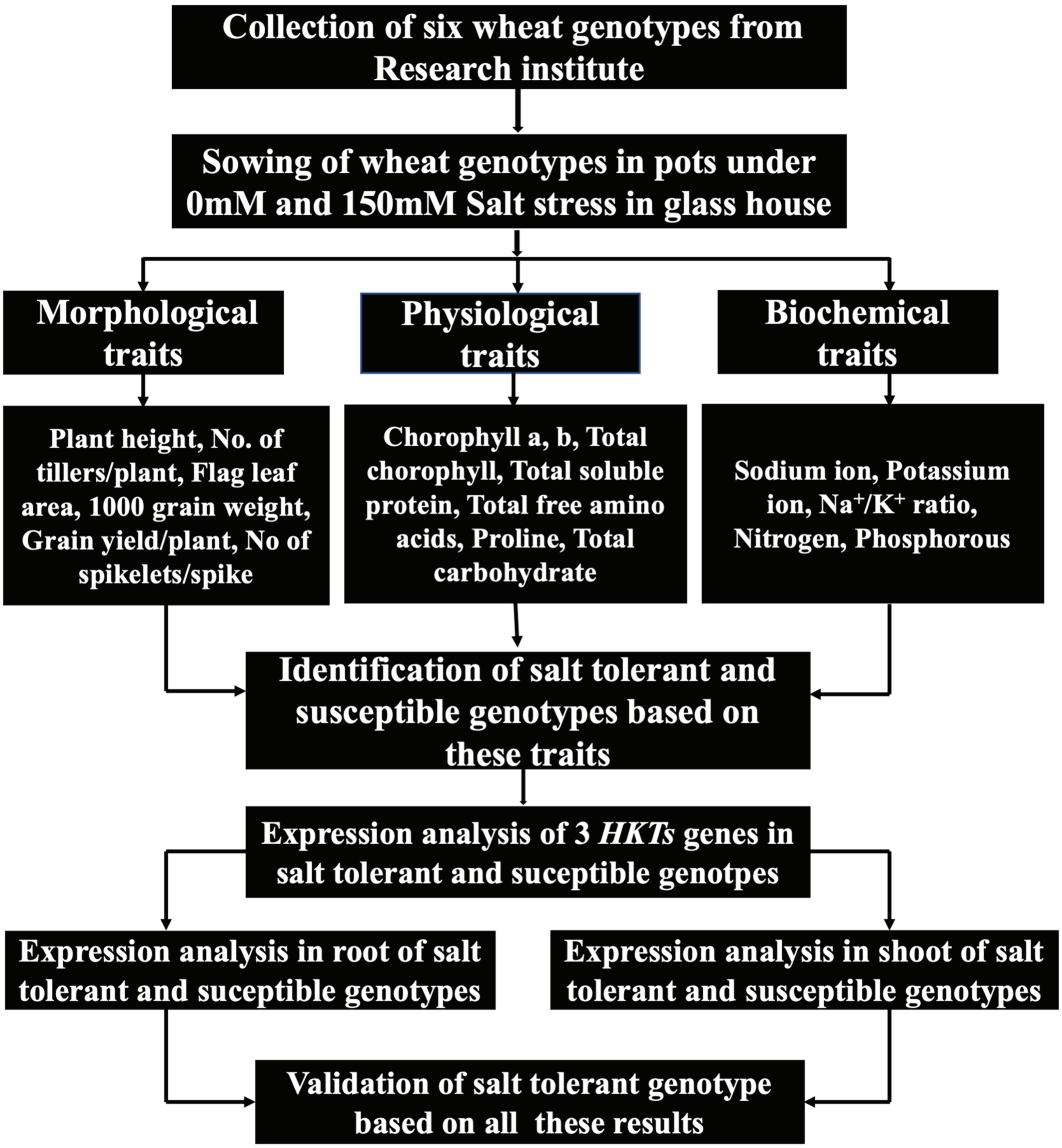
A schematic diagram of research work.

The main objective of this study was to investigate the comprehensive evaluation of wheat genotypes. Six genotypes were used to identify the highly susceptible and highly tolerant genotypes. These wheat genotypes were evaluated by exploiting physiological and biochemical responses under salt stress. Mechanisms responsible for genotype-and tissue-specific differential expression of *TaHKTs* genes were also investigated. The overall sketch of this research is presented in Figure 1.

## Materials and methods

Six wheat genotypes consisting of salt susceptible (Kohistan-97, Fareed-06, A.Sattar) and tolerant (Pasban-90, Inqilab-91, Bakhar-02) wheat (*Triticum aestivum* L.) genotypes were obtained from Regional Agricultural Research Institute, Bahawalpur. The agronomic experiment was conducted in 2019–2020 at Muhammad Nawaz Shareef University of Agriculture, Multan, Pakistan. Seeds of each variety were sterilized in a 5% sodium hypochlorite solution for 15 min and then rinsed with distilled water three times before further experimentation.

Two experiments were conducted at the same time, one experiment was used for seedlings, while the other was for maturity purposes. In the seedling experiment, aluminum trays of 200 × 100 cm size with pores at the bottom were filled with river sand, washed with tap water, and then distilled water. The depth of the sand was 5 inches. Seeds of each cultivar of wheat were allowed to germinate for 1 week. The experiment was arranged in a randomized complete block design with two treatments (0, 150 mM NaCl) and four replicates. Seeds of six wheat varieties were sown in rows at a 15 cm distance from each other. Treatments were started after 1 week of sowing. Salinity concentration was increased stepwise in aliquots of 50 mM to avoid salt shock (Chartzoulakis and Loupassaki, 1997). An adequate amount of water was applied to each tray on alternate days to minimize evapotranspiration loss. The appropriate treatment solution was applied every week to each tray to regularly maintain the salinity level in the sand. After 5 weeks of growth, plants were harvested for studying different morphological and physiological traits.

Similarly, for the maturity experiment, three pots for each genotype were grown under control conditions at a glass house in 2019–2020. The experiment was arranged in Complete Randomized Design (CRD) with two treatments (0 and 150 mM NaCl) in three replicates. Two-week-old wheat seedlings were subjected to salt stress conditions. Salinity concentration was increased stepwise in aliquots of 50 mM to avoid salt shock (Chartzoulakis and Loupassaki, 1997). An adequate amount of water was applied to each pot on alternate days to minimize evapotranspiration loss. The appropriate treatment solution was applied every week to each pot to regularly maintain the salinity level in the sand. To study the effects of salt stress on the growth and development of wheat genotypes, plant height, the number of tillers/plant, flag leaf area, 1,000 grain weight, grain yield/plant, and the number of spikelets/spike were recorded at maturity stage.

### Measurement of growth and development traits

After harvesting 5 weeks’ worth of seedlings, shoot fresh weight and root fresh weight were determined. These samples were then oven dried at 75°C for 3 days with three biological replications and then measured the dried shoot weight and dried root weight in grams. To study the effects of the salt stress on the growth and development of wheat genotypes, plant height, the number of tillers/plant, flag leaf area, 1,000 grain weight, grain yield/plant, and the number of spikelets/spike were recorded at maturity stage. Plant height was measured in centimeters with three biological replications. The number of tillers/plant and spikelets/spike were counted with three replications. The 1,000 grain weight and grain yield/plant were measured in grams.

### Chlorophyll

The chlorophyll content was measured as explained by Withan et al. (1971). Sample (0.1) from the fully matured healthy leaf was homogenized in 4 ml of 80% acetone solution and was diluted up to 8 ml with 80% acetone solution. Optical densities were read at 645 nm and 663 nm using a spectrophotometer (Hitachi U-2000, Tokyo, Japan).


$$\mathrm{Chlorophyll}\;a\;\left(\mathrm{mg}/g\;fwt\right)=\frac{\left[12.7\left(O.D663\right)-2.69\left(O.D645\right)\right]\times V}{1,000\times \mathrm{weight}\left(g\right)}$$

$$\mathrm{Chlorophyll}\;b\;\left(\mathrm{mg}/g\;fwt\right)=\frac{\left[22.9\left(O.D645\right)-4.68\left(O.D663\right)\right]\times V}{1,000\times \mathrm{weight}\left(g\right)}$$

$$\mathrm{Total chlorophyll}\;\left(\mathrm{mg}/g\;fwt\right)=\mathrm{Chlorophyll}\;a+\mathrm{Chlorophyll}\;b$$

V = volume of the extract (ml).

W = weight of the fresh leaf tissue (g).

OD = optical density.

### Total free amino acids

Hamilton and Van Slyke (1943) protocol were used to measure the total free amino acids. The formula used to measure this trait is given here:


$$\begin{array}{l} \mathrm{Total free amino acids}\;\left(\mathrm{mg}/g\right)\\ =\frac{\mathrm{Reading}\;\left(\mathrm{ppm}\right)\times \mathrm{Volume of extract}\times \mathrm{Dilution factor}}{\mathrm{Sample weight}\;\left(g\right)\times 1,000} \end{array}$$

### Total soluble proteins and carbohydrates

Bradford (1976) method was used to measure the total soluble protein. In this method, 0.1 ml of extracted sample was reacted with 5 ml Bradford reagent, and a spectrophotometer was used to take the reading at 595 nm wavelength. For estimation of total soluble protein,


$$\begin{array}{l} \mathrm{Total soluble proteins}\;\left(\mathrm{mg}/g\right)\\ =\frac{\mathrm{Reading}\;\left(\mathrm{ppm}\right)\times \mathrm{Volume of extract}\times \mathrm{Dilution factor}}{\mathrm{Sample weight}\;\left(g\right)\times 1,000} \end{array}$$

Total carbohydrates were determined by using the method described by Hussain et al. (2021).

### Macronutrients (N, P)

The protocol, reported by Allen et al. (1986) was used to measure nitrogen (N) and phosphorous (P). Briefly, the dried and grounded leaves (100 mg) were digested in 2 ml of sulphuric peroxide digestion mixture and obtained a colorless solution.

### Proline content

Ninhydrin reagent was used to measure the proline content by following the method reported by Bates et al. (1973). The following formula was used to measure the proline content:


$$\begin{array}{l} \mathrm{Proline}\left(\mu M/g\;DW\right)\\ =\left[\begin{array}{l} \left(\mathrm{\mu g}\;\mathrm{Proline}/\mathrm{ml}\to \mathrm{ml}\;\mathrm{Toluene}\right)\\ /115.5\mathrm{\mu g}/\mu M \end{array}\right]/\left[\left(g\;\mathrm{sample}\right)/5\right]\text{.} \end{array}$$

### Mineral elements

The measurement of Nitrogen ion and Potassium ion in leaves followed the method of Allen et al. (1986). The leaves were dried and digested in a digestion mixture at 250\u00B0C on the hot plate. Flame photometer was used to measure the Na + and K+.

### Expression analysis

Gene expression analysis was performed for *HKTs* genes from the samples of root and shoot of tolerant and susceptible wheat genotypes. These samples were collected from plants and quickly put in the liquid nitrogen and then shifted to the-800C refrigerator for RNA extraction. The detailed protocol of RNA extraction, cDNA formation, and qRT-PCR followed the method described by Zhang et al. (2019). Similarly, genes and their primer information were obtained from Singh et al. (2015). Three biologicals with two technical replications were used for quantitative expression of these genes by using the 7,500 Fast Real-Time PCR System (Applied Biosystems, Forster City, CA, United States). In these analyses, TaActin was used as a housekeeping gene.

### Statistical analysis

The data were subjected to 3-way ANOVA using a statistical computer package COSTAT (Cohort software, Berkeley, United States). Mean values were compared with the least significant difference test, which was measured in Microsoft Excel.

## Results

### Plant growth

**Figure 2:**
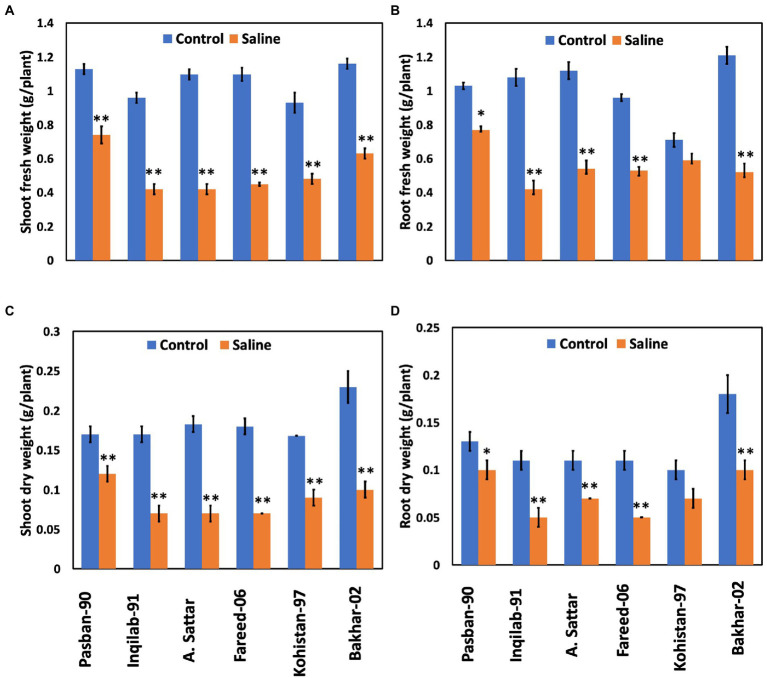
Growth attributes of wheat varieties under salt stress (150 mM) and control conditions. **(A)**, shoot fresh eight **(B)**, root fresh weight **(C)**, shoot dry weight, and **(D)**, root dry weight. Bars indicate ±. Mean values are indicated on the vertical axis. Asterisks (**) indicate that the trait mean was significantly different (value of *p* < 0.01) between the salt and control conditions.

Significant reduction (*p* ≤ 0.001) in shoot and root fresh and dry weight was observed under salt stress conditions (Table 1; Figure 2). The wheat genotypes also showed a negative response to the growth attributes. Shoot fresh and dry weight were reduced to 50% in Inqilab-91, Fareed-06, and A. Sattar under salinity conditions. Similarly, Kohistan-97 and Bakhar-02 showed a 30%–40% reduction in the same saline condition, while little reduction was observed in the biomass of Pasban-90. Moreover, the decline in root fresh and dry biomass ranged from 47% to 52% in Inqilab-91, A. Sattar, and Fareed-06; whereas the reduction in Pasban-90 and Kohistan-97 was 20%–30% for fresh and dry root weight, respectively (Figure 2).

**Table 1:** ANOVA values of the data from growth attributes, yield related attributes, and physiological and biochemical attributes of six varieties when subjected to 150 mM salt stress.

**SOV**	**df**	**Shoot f. wt.**	**Shoot d. wt.**	**Root f. wt.**	**Root d. wt.**	**Chlorophyll a**
Salt	1	2.523^***^	0.108^***^	2.749^***^	0.043^***^	2.570^***^
Var	5	0.087^***^	0.002^***^	0.049^***^	0.001^***^	0.027ns
Salt*var	5	0.024^**^	0.001^***^	0.088^***^	0.001^***^	0.007ns
Total	11	2.634	0.111	2.886	0.045	2.604
**SOV**	**df**	**Chlorophyll a/b**	**Total Chlorophyll**	**Leaf Nitrogen**	**Leaf Phosphorous**	**Amino acids**
Salt	1	6.325^***^	2.581^***^	1,319.138^***^	13.830^***^	10.100^***^
Var	5	0.529ns	0.112ns	23.277^*^	0.304^*^	0.789^*^
Salt*var	5	0.141ns	0.057ns	28.315^*^	0.179ns	1.455^**^
Total	11	6.995	2.75	1,370.766	14.313	12.344
**SOV**	**df**	**Plant height**	**Flag leaf area**	**Grain yield per plant**	**1,000 grain weight**	**Proline**
Salt	1	484.33^***^	72.12^**^	60.123^***^	0.991^**^	116.76^***^
Var	5	9,021.34^***^	312.43^**^	323.14^***^	37.077^**^	1,175.12^***^
Salt*var	5	190.42^**^	122.491^**^	69.786^***^	0.673^**^	119.876^***^
**SOV**	**df**	**Shoot Na**^**+**^	**Shoot K**^**+**^	**Chlorophyll b**	**Total proteins**	**Total carbohydrate**
Salt	1	29.987^***^	26.556^***^	0.261^***^	6.671ns	92.098^***^
Var	5	4,300.12^***^	1,458.13^***^	0.002ns	11.493^**^	20,342.811^***^
Salt*var	5	28.125^***^	6.132^***^	0.001ns	36.491^***^	159.98^***^

ns, non-significant; SOV, source of variation; df, degree of freedom. ^*, **, ***^significant at 0.05, 0.01, and 0.001 probability.

### Yield related traits

**Figure 3:**
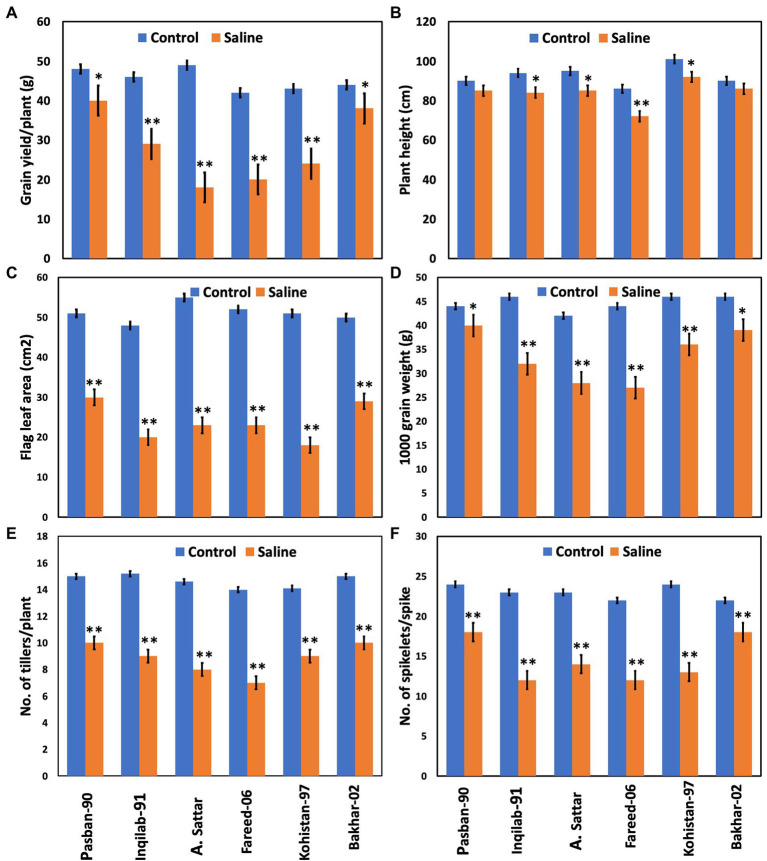
Yield attributes of wheat varieties at the maturity stage under salt stress (150 mM) and control condition (0 mM). **(A)** Grain yield/plant, **(B)** plant height, **(C)** flag leaf area, **(D)** 1,000 grain weight, **(E)** no. of tillers/plant, **(F)** no. of spikelet’s/spike. Bars indicate ±. Mean values are indicated on the vertical axis. Asterisks (**) indicate that the trait mean was significantly different (value of *p* < 0.01) between the salt and control conditions.

Salt stress significantly affected the yield and yield-related traits in the present study (Table 1; Figure 3). All the wheat genotypes differed in their yield components under salt stress. Grain yield/plant and 1,000 grain weight of Pasban-90 and Bakhar-02 were higher under salt stress conditions compared to the other four wheat genotypes. Similarly, the number of spikelets per spike also decreased in all four salt sensitive wheat genotypes (Inqilab-91, A. Sattar, Fareed-06, and Kohistan-97). Based on these results, the number of tillers/plant were maximum in salt tolerant wheat material (Pasban-90 and Bakhar-02), compared to salt sensitive wheat material in which there was a reduction in the number of tillers/plant. A significant difference in plant height was observed in wheat germplasm under salt stress conditions. Maximum reduction was observed in Fareed-06 and Kohistan-97 followed by A. Sattar and Inqilab-91. While the minimum reduction in plant height was observed in the Pasban-90 and Bakhar-02 under salinity stress. Salt stress had a significant impact on the flag leaf area. In total, there was a 60%–70% reduction in flag leaf area observed in the Inqilab-91, A. Sattar, Fareed-06, and Kohistan-97, whereas there was a 30%–40% decrease in the Pasban-90 and Bakhar-02, as shown in Figure 3.

### Photosynthetic pigment and other physiological traits

**Figure 4:**
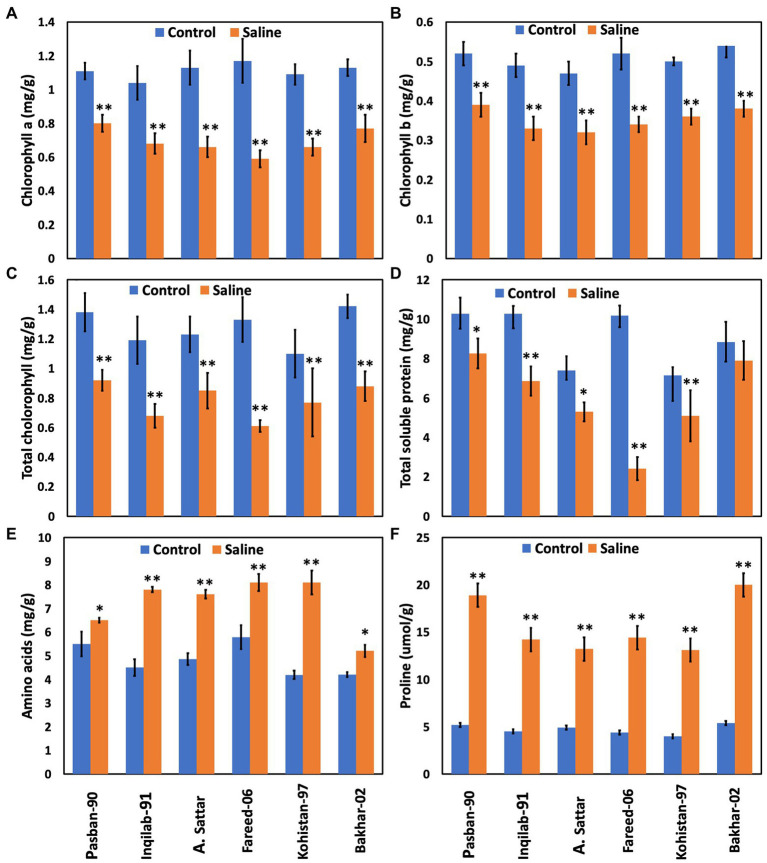
Physiological attributes of six wheat varieties under salt stress (150 mM) and control condition (0 mM). **(A)** Chlorophyll a, **(B)** chlorophyll b, **(C)** total Chlorophyll, **(D)** total soluble protein, **(E)** total free amino acids, **(F)** proline. Bars indicate ±. Mean values are indicated on the vertical axis. Asterisks (**) indicate that the trait mean was significantly different (value of *p* < 0.01) between the salt and control conditions.

Chlorophyll a, b, and total chlorophyll contents significantly decreased due to salt stress in all studied wheat genotypes. Reduction of chlorophyll a, b, and total chlorophyll contents was observed in the A. Sattar, Fareed-06, Kohistan-097, and Inqilab-91. However, chlorophyll a, b, and total chlorophyll were reduced in the Pasban-90 and Bakhar-02 (Figure 4). We observed a significant reduction in the total soluble protein, however, in all wheat genotypes there was a significant increase in the total free amino acids calculated as compared to normal conditions. Furthermore, a reduction in total soluble proteins and increased amino acids was observed in salt tolerant genotypes (Pasban-90 and Bakhar-02). Fareed-06, A. Sattar, and Kohistan had lower total soluble protein in salt stress conditions. Additionally, the maximum amino acids observed in the Fareed-06 while minimum amino acids were observed in Pasban-90 under salt stress. Similarly, the accumulation of proline in the leaves of all wheat varieties significantly increased in salt stress conditions. Maximum proline was observed in Pasban-90 and Bakhar-02 while less accumulation was observed in Kohistan-97 and Fareed-06 (Figure 4).

### Relative distribution of inorganic solutes and total carbohydrates

**Figure 5:**
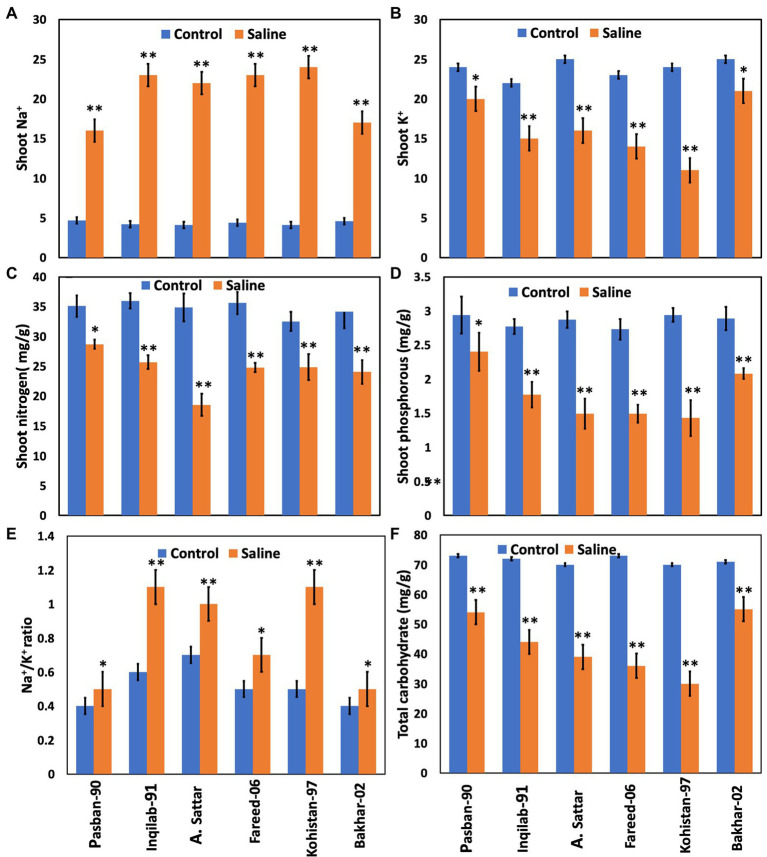
Biochemical attributes and total carbohydrates of six wheat varieties under salt stress (150 mM) and control condition (0 mM). **(A)** Shoot Na^+^, **(B)** shoot K^+^, **(C)** shoot nitrogen, **(D)** shoot phosphorous, **(E)** Na^+^/K^+^ ratio, and **(F)** total carbohydrate. Bars indicate ±. Means values are indicated on the vertical axis. Asterisks (**) indicate that the trait mean was significantly different (value of *p* < 0.01) between the salt and control conditions.

The concentration of Na^+^ increased significantly whereas, K^+^, Phosphorous, and nitrogen accumulation significantly decreased in all wheat genotypes under salt stress. The accumulation in sensitive genotypes was higher than salt tolerant genotypes (Pasban-90 and Bakhar-02). Kohistan-97 accumulated the highest level of Na^+^ in salt stress conditions (Figure 5). Similarly, Pasban-90 accumulated higher K^+^ as compared to control, and Kohistan-97 accumulated less K^+^ in salt stress conditions. Both N and P accumulation in leaves were decreased in all wheat varieties. Nitrogen and phosphorous accumulation were observed higher in Pasban-90 and Bakhar-02. A significant increase in the Na^+^/K^+^ ratio was calculated in all wheat varieties. The Na^+^/K^+^ ratio was lower in Pasban-90 but higher in Kohistan-97 (Figure 5).

### Expression analysis of salt tolerant and susceptible varieties

**Figure 6:**
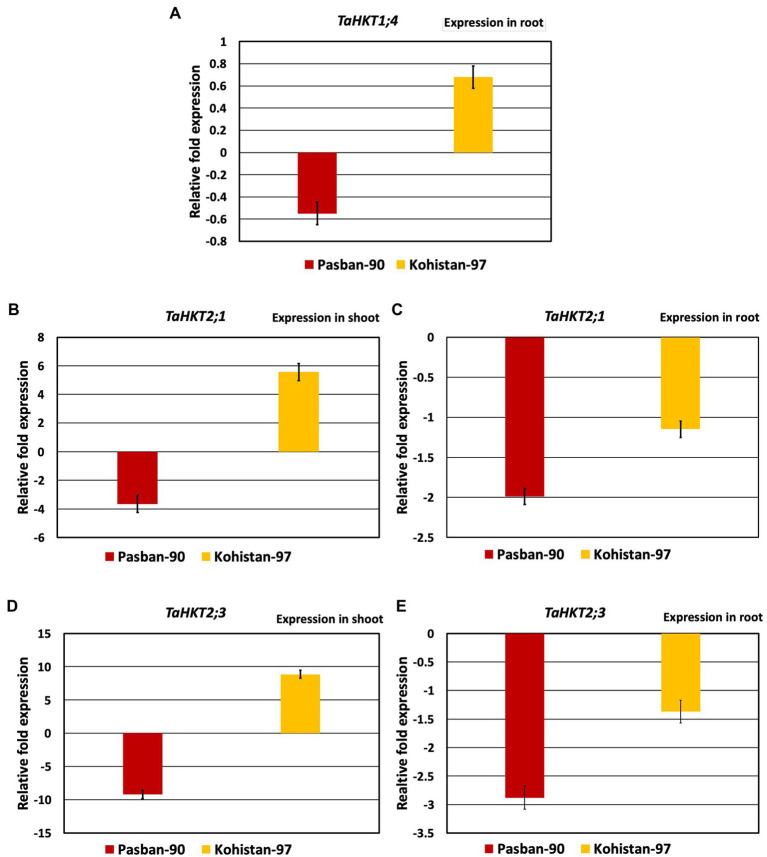
High affinity potassium transporter (HKT) salt responsive genes expression analysis (RT-qPCR). **(A)** Accumulation of transcript of TaHKT1;4 in root, **(B)** expression analysis of TAHKT2;1 in shoot, **(C)** expression analysis of TaHKT2;1 in root, **(D)** expression analysis of TaHKT2;3 in shoot, **(E)** expression analysis of TYaHKT2;3 in root. The results are mean fold change in relative expression over the control with three biological and three technical replicates, normalized with actin (reference) gene expression. Bars represent standard deviation.

Based on the morpho-physiological and biochemical analysis, two genotypes were selected for expression analysis. Pasban-90 was selected as highly salt tolerant and Kohistan-97 was selected as susceptible to salt stress. Three *HKT* genes were identified to investigate the effect of salt stress on transcription of HKTs and wheat genotypes correlation ability. *TaHKT1; 4* showed root specific expression, as reported in previous studies (Kumar et al., 2017) *TaHKT2; 1* and *TaHKT2; 3* showed expression both in the shoot and root of the plants. In Pasban-90, *TaHKT1; 4* downregulated in roots under salt stress conditions while it showed upregulation for Kohistan-97 (Figure 6). Similarly, the other two genes (*TaHKT2; 1* and *TaHKT2; 3*) are expressed differently in shoot and root under salt stress conditions in both genotypes. Expression analysis of *TaHKT2; 1* revealed that it was downregulated for Pasban-90 in the shoot while, showed positive regulation for Kohistan-97 whereas in roots, this gene showed downregulation for both genotypes (Figure 6). The expression of *TaHKT2; 3* was similar to *TaHKT2; 1* in shoots with downregulation for Pasban-90 and upregulation for Kohistan-97 under salt stress. Similarly, this gene (*TaHKT2; 3*) was downregulated for both genotypes under salt stress in roots.

## Discussion

Considerable efforts have been made over the last two decades to identify salt tolerant varieties in different crops by utilizing physiological, biochemical, and genetic approaches, but still, little progress has been made in this regard. Our literature review suggested that with the use of these physiological and biochemical parameters, researchers will be able to develop salt tolerant genotypes for wheat (Zuo et al., 2021; Zhao et al., 2022). In this study, six genotypes were used that were initially screened by the Regional Agricultural Research Institute from their germplasm. According to the data, three genotypes were susceptible and three were salt tolerant.

The imposition of salt stress in the early stage and observation of plant biomass helps to evaluate the salt tolerant genotypes in wheat (Ai-Ke et al., 2009). Change in the root and shoot biomass after salt stress in wheat genotypes helps evaluate salt tolerant varieties. Therefore, in this study, a significant reduction in biomass of Fareed-06 and Kohistan-97 was observed. Under saline conditions, less reduction in biomass or crop yield will determine the salt tolerance variety (Hussain et al., 2021). The yield of a wheat crop depends upon many yield-related traits such as grain, numbers, and the size of grains. Salt tolerant genotypes such as Pasban-50 and Bakhar-02 in the present study had higher yields under salt stress as compared to other salt sensitive genotypes. Similarly, there was a significant difference in the yield and number of grains due to genotypic differences under salt stress. Wheat grain numbers per plant mainly depend on tillering capacity and the number of grains per spike. Similarly, the grain size of wheat depends upon the photosynthetic activity of the plant (Mansour et al., 2020). A significant decrease in the number of tillers/plant and grain yield was observed in the present study. Likewise, a decline in grain size is another important agronomic trait contributing to the reduction of wheat yield under saline conditions. This can be explained by salt stress reducing the availability of photo-assimilates or reducing the translocation of photo-assimilates from the source to the developing grains. According to results, salt tolerant genotypes possessed the ability to produce maximum tillers with less abortion of florets in spikelets and also had a better ability to translocate photo-assimilates to grains from leaves (Arfan et al., 2007).

Salinity stress affects chlorophyll by reducing the content and overall photosynthesis process (Ashraf, 2004). There was no significant reduction of Chlorophyll a, b and total chlorophyll content in Pasban-50 and Bakhar-02 while a significant reduction was observed in other genotypes. Therefore, these results supported the idea that Pasban-50 is a salt tolerant variety. Whereas less chlorophyll content was observed in Kohistan-97 resulting in a salt sensitive variety. This type of salt effect on chlorophyll content has already been observed in cabbage and other plant species (Mansour et al., 2016). Chlorophyll synthesis and the photosynthesis process are adversely affected by the accumulation of Na^+^ in plants (Jiang et al., 2017). Proline plays an important role in osmotic adjustment and the stability of the structure during stress in plants (Romero-Aranda et al., 2006). According to different studies, cellular machinery releases some solutes that help to maintain the redox potential in plants under stress. Proline can enhance salt tolerance by protecting the cell membrane and enzymes (Munns, 2002). Similarly, a higher accumulation of total free amino acid in wheat varieties could not help to maintain the plant water status and thus, created adverse osmotic effects under salt stress (Azeem et al., 2019). Our results were similar to the findings observed in different crops such as the safflower and brassica species (Platten et al., 2006; Rauf et al., 2010). Accumulation of stress responsive proteins play a significant role during salt stress. In this study, lower total soluble protein and an increase in free amino acids were observed which indicated that salt stress decreases the proteins by activating the proteases. This eventually helps to increase the total free amino acids (Sairam et al., 2002). Therefore, salt tolerant varieties with relatively high total soluble proteins, compared to salt sensitive varieties, provide information on the total proteins in a salt tolerant variety (Ashraf, 2004).

In plants, shoots and roots showed differential responses to the monovalent and divalent cations in relation to accumulation/distribution. Roots absorb more Na^+^ because it has direct contact with the soil as compared to shoots under control condition. However, in stress conditions Na^+^ is transported and executed from leaves to maintain the optimum level as observed in Pasban-50 and Bakhar-02. Salt-tolerant genotypes possess exclusion mechanisms (regulated expression of *HKTs*) that control the entry of Na^+^ into roots (Munns et al., 2020), and exclusion of the excessive Na^+^ from photosynthetic tissues (Tabassum et al., 2021). The adverse effect of salt stress on K^+^ uptake could be seen in the wheat genotypes where the maximum reduction in absorption/transport was observed. Our findings suggest that salt-tolerant genotypes retain selectivity for K^+^ over Na^+^ and maintain a lower Na^+^/K^+^ ratio under the stress, while the salt-sensitive genotypes failed to do so. There were differential effects in the uptake of nitrogen and phosphorous content. However, this impact had been observed less in salt tolerant varieties, especially in Pasban-50. Similar results have already been observed in previous studies, which outline that salt tolerance characteristics are generally associated with salt exclusion and maintenance of the Na^+/^K^+^ ratio (Zhang et al., 2014). Based on these analyses, Pasban-50 is a salt tolerant genotype because it showed a high level of chlorophyll, proline, and total proteins, and also maintained a low Na^+^/K^+^ ratio; whereas, based on these results, Kohistan-97 is the most sensitive variety (Zhao et al., 2022).

In plants, salt stress could impose many morphological, physiological, biochemical, and several other genetic changes that could be analyzed through expression analysis. Due to this, in this study, expression analysis of three *HKT* genes was performed as it could play an important role in the transportation of Na^+^ and or K^+^. In Pasban-90, *TaHKT1; 4* was root specific (Kumar et al., 2017) and downregulated in stress conditions, therefore, restricting the entrance of Na^+^ in root cells. However, on the other side, this gene was upregulated in Kohistan-97 due to the accumulation of Na^+^ in the root’s cells. Kohistan-97 up-regulation may be the reason for its salt sensitive nature. It has been reported that *AtHKT1* directed the retrieval of Na^+^ from the xylem and its loading into root vacuoles. At a cellular level, Na^+^ homeostasis is regulated by controlling Na^+^ entry into root cells, transporting Na^+^ out of shoot cells, and compartmentalizing Na^+^ into vacuoles (Shi et al., 2003). The other two genes also showed differential expression in the roots and shoots of wheat genotypes under stress conditions. These genes were more downregulated in Pasban-50 as compared to Kohistan-97 in the root. Hence, it can be concluded that the expression of *HKT2; 3* in the roots was weakly correlated to the Na^+^ uptake in Pasban-50. Na^+^ exclusion can also be achieved by the net unloading of xylem by parenchyma cells in the stele (Munns et al., 2006), which could be correlated with the least accumulation of Na^+^ in the shoots of Pasban-50 under the stress. In durum wheat *TaSOS1* differential expression associated with Na^+^ from shoot to root has already been reported by Brini et al. (2009).

## Conclusion

Based on the comprehensive analysis of agronomic, physiological, and biochemical traits, we identified contrasting varieties in relation to salt responses. Chlorophyll content, proline content, and Na^+^/K^+^ ratio indicated that Pasban-50 is a salt tolerant genotype. Moreover, the accumulation of total free amino acids plays a role in changes in osmotic potential but did not play a role in differential salt tolerance. On the other hand, Kohistan-97 was found to be a salt sensitive variety. Expression analysis of *HKTs* also confirms the response of salt tolerant genotypes. A better understanding of the structural, functional, and regulatory mechanisms of *HKTs* will enable us to further improve salt tolerance in plants and develop highly salt tolerant varieties.

## Data availability statement

The raw data supporting the conclusions of this article will be made available by the authors, without undue reservation.

## Author contributions

GS and ZK conceptualized the idea. AI and RIA did experimentation and wrote first draft of the manuscript. SU, FA, and MAS provided technical support in genetics and molecular work. MZA and MKA supported in data analysis. MMH and SHQ reviewed the final draft for improvement. GS and ZK made final revisions before submission. All authors contributed to the article and approved the submitted version.

## Conflict of interest

The authors declare that the research was conducted in the absence of any commercial or financial relationships that could be construed as a potential conflict of interest.

## Publisher’s note

All claims expressed in this article are solely those of the authors and do not necessarily represent those of their affiliated organizations, or those of the publisher, the editors and the reviewers. Any product that may be evaluated in this article, or claim that may be made by its manufacturer, is not guaranteed or endorsed by the publisher.

## References

[ref1] Ai-Ke B. Zheng-Gang G. Hong-Fei Z. Suo-Min W. (2009). A procedure for assessing the salt tolerance of lucerne (*Medicago sativa* L.) cultivar seedlings by combining agronomic and physiological indicators. New Zealand J. Agric. Res. 52, 435–442. doi: 10.1080/00288230909510525

[ref2] Allen S. E. Grimshaw H. M. Rowland A. P. (1986). “Methods in plant ecology” in Chemical Analysis. 2nd Edn. eds. Moore P. D. Chapman S. B. (Oxford: Blackwell Scientific Publication), 285–344.

[ref3] Arfan M. Athar H. R. Ashraf M. (2007). Does exogenous application of salicylic acid through the rooting medium modulate growth and photosynthetic capacity in two differently adapted spring wheat cultivars under salt stress? J. Plant Physiol. 164, 685–694. doi: 10.1016/j.jplph.2006.05.010, PMID: 16884826

[ref4] Arzani A. (2008). Improving salinity tolerance in crop plants: a biotechnological view. In Vitro Cell.Dev.Biol. Plant 44, 373–383. doi: 10.1007/s11627-008-9157-7

[ref5] Arzani A. Ashraf M. (2016). Smart engineering of genetic resources for enhanced salinity tolerance in crop plants. Crit. Rev. Plant Sci. 35, 146–189. doi: 10.1080/07352689.2016.1245056

[ref6] Ashraf M. (2004). Some important physiological selection criteria for salt tolerance in plants. Flora Morphol. Distrib. Funct. Ecol. Plants 199, 361–376. doi: 10.1078/0367-2530-00165

[ref7] Ashraf M. O’Leary J. W. (1995). Distribution of cations in leaves of salt-tolerant and salt-sensitive lines of sunflower under saline conditions. J. Plant Nutr. 18, 2379–2388. doi: 10.1080/01904169509365072

[ref8] Athar H. R. Ashraf M. (2009). “Strategies for crop improvement against salinity and drought stress: An overview,” in Salinity and Water Stress Tasks for Vegetation Science. eds. Ashraf M. Ozturk M. Athar H. R. (Dordrecht: Springer), 1–16.

[ref9] Azeem M. Qasim M. Waseem Abbasi M. Tayyab T. Sultana R. Yousuf Adnan M.. (2019). Salicylic acid seed priming modulates some biochemical parameters to improve germination and seedling growth of salt stressed wheat (*Triticum aestivum* L.). Pak. J. Bot. 51, 1–7. doi: 10.30848/PJB2019-2(1)

[ref10] Bates L. S. Waldren R. P. Teare I. D. (1973). Rapid determination of free proline for water-stress studies. Plant Soil 39, 205–207. doi: 10.1007/BF00018060

[ref11] Bradford M. M. (1976). A rapid and sensitive method for the quantitation of microgram quantities of protein utilizing the principle of protein-dye binding. Anal. Biochem. 72, 248–254. doi: 10.1006/abio.1976.9999, PMID: 942051

[ref12] Brini F. Amara I. Feki K. Hanin M. Khoudi H. Masmoudi K. (2009). Physiological and molecular analyses of seedlings of two Tunisian durum wheat (*Triticum turgidum* L. subsp. durum [Desf.]) varieties showing contrasting tolerance to salt stress. Acta Physiol. Plant. 31, 145–154. doi: 10.1007/s11738-008-0215-x

[ref13] Chartzoulakis K. S. Loupassaki M. H. (1997). Effects of NaCl salinity on germination, growth, gas exchange and yield of greenhouse eggplant. Agric. Water Manag. 32, 215–225. doi: 10.1016/S0378-3774(96)01276-0

[ref14] Hamamoto S. Horie T. Hauser F. Deinlein U. Schroeder J. I. Uozumi N. (2015). HKT transporters mediate salt stress resistance in plants: from structure and function to the field. Curr. Opin. Biotechnol. 32, 113–120. doi: 10.1016/j.copbio.2014.11.025, PMID: 25528276

[ref15] Hamilton P. B. Van Slyke D. D. (1943). The gasometric determination of free amino acids in blood filtrates by the ninhydrin-carbon dioxide method. J. Biol. Chem. 150, 231–250. doi: 10.1016/S0021-9258(18)51268-0

[ref16] Horie T. Hauser F. Schroeder J. I. (2009). HKT transporter-mediated salinity resistance mechanisms in arabidopsis and monocot crop plants. Trends Plant Sci. 14, 660–668. doi: 10.1016/j.tplants.2009.08.009, PMID: 19783197 PMC2787891

[ref17] Hussain N. Ghaffar A. Zafar Z. U. Javed M. Shah K. H. Noreen S.. (2021). Identification of novel source of salt tolerance in local bread wheat germplasm using morpho-physiological and biochemical attributes. Sci. Rep. 11:10854. doi: 10.1038/s41598-021-90280-w, PMID: 34035371 PMC8149405

[ref18] Impa S. M. Sunoj V. S. J. Krassovskaya I. Bheemanahalli R. Obata T. Jagadish S. V. K. (2019). Carbon balance and source-sink metabolic changes in winter wheat exposed to high night-time temperature. Plant Cell Environ. 42, 1233–1246. doi: 10.1111/pce.13488, PMID: 30471235

[ref19] Jiang C. Zu C. Lu D. Zheng Q. Shen J. Wang H.. (2017). Effect of exogenous selenium supply on photosynthesis, Na+ accumulation and antioxidative capacity of maize (*Zea mays* L.) under salinity stress. Sci. Rep. 7, 42039. doi: 10.1038/srep42039, PMID: 28169318 PMC5294586

[ref20] Kiani R. Arzani A. Mirmohammady Maibody S. A. M. (2021). Polyphenols, flavonoids, and antioxidant activity involved in salt tolerance in wheat, aegilops cylindrica and their amphidiploids. Front. Plant Sci. 12:646221. doi: 10.3389/fpls.2021.646221, PMID: 33841475 PMC8027307

[ref21] Kumar S. Beena A. S. Awana M. Singh A. (2017). Salt-induced tissue-specific cytosine methylation downregulates expression of *HKT* genes in contrasting wheat (*Triticum aestivum* L.) genotypes. DNA Cell Biol. 36, 283–294. doi: 10.1089/dna.2016.3505, PMID: 28384069 PMC5385449

[ref22] Mansour H. A. Abd El-Hady M. Bralts V. F. Engel B. A. (2016). Performance automation controller of drip irrigation systems using saline water for wheat yield and water productivity in Egypt. J. Irrig. Drain. Eng. 142:015016005. doi: 10.1061/(ASCE)IR.1943-4774.0001042

[ref23] Mansour E. Moustafa E. S. A. Desoky E.-S. M. Ali M. M. A. Yasin M. A. T. Attia A.. (2020). Multidimensional evaluation for detecting salt tolerance of bread wheat genotypes under actual saline field growing conditions. Plan. Theory 9, 1324. doi: 10.3390/plants9101324, PMID: 33036311 PMC7601346

[ref24] Marcińska I. Czyczyło-Mysza I. Skrzypek E. Filek M. Grzesiak S. Grzesiak M. T.. (2013). Impact of osmotic stress on physiological and biochemical characteristics in drought-susceptible and drought-resistant wheat genotypes. Acta Physiol. Plant. 35, 451–461. doi: 10.1007/s11738-012-1088-6

[ref25] Munns R. (2002). Comparative physiology of salt and water stress. Plant Cell Environ. 25, 239–250. doi: 10.1046/j.0016-8025.2001.00808.x 11841667

[ref26] Munns R. James R. A. Läuchli A. (2006). Approaches to increasing the salt tolerance of wheat and other cereals. J. Exp. Bot. 57, 1025–1043. doi: 10.1093/jxb/erj100, PMID: 16510517

[ref27] Munns R. Passioura J. B. Colmer T. D. Byrt C. S. (2020). Osmotic adjustment and energy limitations to plant growth in saline soil. New Phytol. 225, 1091–1096. doi: 10.1111/nph.15862, PMID: 31006123

[ref28] Platten J. D. Cotsaftis O. Berthomieu P. Bohnert H. Davenport R. J. Fairbairn D. J.. (2006). Nomenclature for HKT transporters, key determinants of plant salinity tolerance. Trends Plant Sci. 11, 372–374. doi: 10.1016/j.tplants.2006.06.001, PMID: 16809061

[ref500] Rauf S. da Silva J. T. Khan A. A. Naveed A. (2010). Consequences of plant breeding on genetic diversity. Intl. J. Plant Breed 4, 1–21., PMID: 28384069

[ref29] Romero-Aranda M. R. Jurado O. Cuartero J. (2006). Silicon alleviates the deleterious salt effect on tomato plant growth by improving plant water status. J. Plant Physiol. 163, 847–855. doi: 10.1016/j.jplph.2005.05.010, PMID: 16777532

[ref30] Sairam R. K. Rao K. V. Srivastava G. C. (2002). Differential response of wheat genotypes to long term salinity stress in relation to oxidative stress, antioxidant activity and osmolyte concentration. Plant Sci. 163, 1037–1046. doi: 10.1016/S0168-9452(02)00278-9

[ref31] Saqib M. Zörb C. Rengel Z. Schubert S. (2005). The expression of the endogenous vacuolar Na+/H+ antiporters in roots and shoots correlates positively with the salt resistance of wheat (*Triticum aestivum* L.). Plant Sci. 169, 959–965. doi: 10.1016/j.plantsci.2005.07.001

[ref32] Shi H. Lee B. Wu S.-J. Zhu J.-K. (2003). Overexpression of a plasma membrane Na+/H+ antiporter gene improves salt tolerance in *Arabidopsis thaliana*. Nat. Biotechnol. 21, 81–85. doi: 10.1038/nbt766, PMID: 12469134

[ref33] Singh A. Bhushan B. Gaikwad K. Yadav O. P. Kumar S. Rai R. D. (2015). Induced defense responses of contrasting bread wheat genotypes under differential salt stress imposition. Indian J. Biochem. Biophys. 52, 75–85. 26040114

[ref34] Tabassum J. Ahmad S. Hussain B. Mawia A. M. Zeb A. Ju L. (2021). Applications and potential of genome-editing Systems in Rice Improvement: current and future perspectives. Agronomy 11:1359. doi: 10.3390/agronomy11071359

[ref35] Withan F. H. Blaydes Devlin R. M., (1971). Experiments in Plant Physiology. Van Nostrand Reinhold Co., New York, NY, 55–58.

[ref36] Zhang S. Guo H. Irshad A. Xie Y. Zhao L. Xiong H.. (2019). The synergistic effects of TaAGP.L-B1 and TaSSIVb-D mutations in wheat lead to alterations of gene expression patterns and starch content in grain development. PLoS One 14:e0223783. doi: 10.1371/journal.pone.0223783, PMID: 31603940 PMC6788705

[ref37] Zhang L. Ma H. Chen T. Pen J. Yu S. Zhao X. (2014). Morphological and physiological responses of cotton (*Gossypium hirsutum* L.) plants to salinity. PLoS One 9:e112807. doi: 10.1371/journal.pone.0112807, PMID: 25391141 PMC4229235

[ref38] Zhao Y. Zhang F. Mickan B. Wang D. Wang W. (2022). Physiological, proteomic, and metabolomic analysis provide insights into bacillus sp.-mediated salt tolerance in wheat. Plant Cell Rep. 41, 95–118. doi: 10.1007/s00299-021-02788-0, PMID: 34546426

[ref39] Zuo Z. Ye F. Wang Z. Li S. Li H. Guo J.. (2021). Salt acclimation induced salt tolerance in wild-type and chlorophyl b-deficient mutant wheat. Plant Soil Environ. 67, 26–32. doi: 10.17221/429/2020-PSE

